# Relationship between GPS-based community mobility data and orthopedic trauma admissions during the COVID-19 pandemic in Austria: a multicenter analysis

**DOI:** 10.1007/s00508-024-02420-7

**Published:** 2024-08-26

**Authors:** Natasa Jeremic, Harald Kurt Widhalm, Kevin Doering, Domenik Popp, Matthias Stark, Cornelia Ower, Arora Rohit, Roberto Boesenberg, Andreas Leithner, Arastoo Nia

**Affiliations:** 1 Department of Orthopedics and Trauma Surgery, Division of Trauma Surgery, Medical University of Vienna, Währinger Straße 18–20, 1090 Vienna, Austria; 2 Department of Orthopedic Surgery and Trauma Surgery, Medical University of Innsbruck, Innsbruck, Austria; 3 Department of Orthopedics and Trauma, Medical University of Graz, Graz, Austria; 4 Department of Orthopedics and Trauma, LKH Neunkirchen, Neunkirchen, Austria; 5 Vienna University of Economics and Business, Vienna, Austria

**Keywords:** COVID-19, Orthopedic trauma, GPS-based, Mobility, Google

## Abstract

**Objective:**

The main objective of this study was to examine the relationship between mobility patterns during the coronavirus disease 2019 (COVID-19) pandemic and orthopedic trauma patients in Austria. Utilizing global positioning system (GPS)-based mobility data, the attempt was to assess both the impact of COVID-19 lockdowns on reducing orthopedic trauma patients and the degree of compliance to the imposed movement restrictions.

**Methods:**

This retrospective analysis included all patients (283,501) treated at 3 major level I trauma centers in Austria. Analyzed time periods were 1 January 2019 to 8 February 2021. Freely available GPS-based mobility data from Google and Apple Inc. was gathered.

**Results:**

A moderate to strong correlation between the cumulative average outpatients and the assessed mobility index was observed for all cities (Google: r = 0.70 *p* < 0.001, 95% confidence interval, CI: 0.67–0.73; Apple: r = 0.64 *p* < 0.001, 95% CI: 0.61–0.67). A significant linear regression equation was found for Vienna (adjusted r^2^ = 0.48; F(1, 350) = 328,05; *p* < 0.01). During the first lockdown there was a drastic decline in mobility (up to −75.36%) and in numbers of orthopedic trauma outpatients (up to −64%, from 153 patients/day 2019 to 55 patients/day 2020) in comparison to the prepandemic era. The decline diminished as time passed.

**Conclusion:**

Analyses of GPS-based mobility patterns show a correlation with trauma patient numbers. These findings can be used to develop prediction models, leading to better resource planning and public health policy, enhancing patient care and cost-effectiveness, especially in the event of future pandemics. Furthermore, the results suggest that compliance to mobility restrictions decreased over time during the COVID-19 pandemic, resulting in increased mobility and trauma patients.

## Introduction

During the coronavirus disease 2019 (COVID-19) pandemic healthcare systems have faced increased demands and challenges globally [1]. After the first COVID-19 lockdown in Austria in early 2020, several orthopedic trauma surgery departments analyzed their epidemiological data and tried to define guidelines for optimal patient care and hygienic measures to ensure improvement of patient care for following infection waves [2]. Hospitals have struggled to manage the increase in patient numbers while also maintaining COVID-19 protocols, leading to longer waiting times and increased pressure on healthcare staff and allocation of resources [3]. When further lockdowns emerged due to novel infection peaks one governmental directive has been to reduce the populations’ mobility by imposing movement restrictions. Besides avoiding further disease spread, a wider goal of the movement restrictions was the reduction of accidents in order to preserve intensive care beds [4]. Trauma patients place a significant burden on healthcare systems [5]. The ability to accurately predict the number of trauma patients would be crucial in ensuring that healthcare resources are optimally allocated, and that patient care is delivered efficiently and effectively, particularly in exceptional situations such as a pandemic.

Global positioning system (GPS)-based data are already widely used in retail, tourism, and economy [6]. Especially since the first COVID-19 outbreak, the data gained further interest in a medical view [7]. Multiple mobility data analyses suggested a decrease of compliance to governmentally imposed measures with time passing [8]; however, information on the relationship between mobility data and the number of trauma patients is still sparse. The aim was to validate the assumption that a reduction of the population’s mobility leads to a reduction in trauma patients and to further assess the feasibility of GPS-based mobility reports as a prediction tool for trauma patient loads. Thus, the study aimed to answer following questions: 1) is there a relevant relationship between GPS data and the number of trauma patients? 2) How did the epidemiology of orthopedic trauma admissions in Austria and the degree of compliance to the movement restriction evolve during the subsequent lockdowns?

## Material and methods

To estimate a countrywide trend, anonymized patient data from three large hospitals across Austria were collected retrospectively. It should be noted that these three cities do not necessarily represent the whole of Austria, and therefore the findings may not be fully generalizable to more rural regions of the country. The participating centers comprised the University Hospital of Vienna, the University Hospital of Graz as well as the University Hospital of Innsbruck.

## Main outcomes and measures

The primary study outcome before collection of the data was to evaluate the effectiveness of the imposed measures and to assess the impact of the COVID-19 lockdowns on orthopedic trauma services in Austria.

## Patient selection, timeframes and parameters

All outpatients admitted to the participating trauma departments in the period from 01/01/2019 to 08/02/2021 were included. Time intervals of special interest were all periods where lockdowns were imposed in Austria. There were three major hard lockdowns during the observed time period (first lockdown: 15/03/2020 to 30/04/2020, second lockdown: 17/11/2020 to 06/12/2020, third lockdown: 26/12/2020 to 07/02/2021). Each lockdown differed in rigorousness of the restrictions. It was decided to choose the end of the 3rd Austrian lockdown as an endpoint of the time period, as it was the last state-consistent lockdown and marked the end of the time where no vaccination against COVID-19 was available for the populace. As there are studies suggesting a change in mobility patterns of people after getting vaccinated, the bias within this study was therefore eliminated by choosing a timeframe before vaccinations were available.

## Mobility data

For the purpose of estimating the populations movement, the publicly available data from Google’s community mobility reports (Google LLC, Mountain View, CA, USA) and Apple mobility trend data (Apple Inc., Cupertino, CA, USA) were utilized. The data are collected from users that enabled the usage of their location data on their mobile devices. The mobility data from Google and Apple is completely anonymized and does not provide any possibility to trace back certain users. Google mobility reports display the change of visits to certain categorized places compared to baseline days, which are calculated as the median value of each weekday in the period of 03/01/2020 to 06/02/2020 [9]. Analogous to this, Apple’s data show the relative amount of direction requests compared to the baseline amount on 13/01/2020 [10]. In order to compare the number of outpatients and the mobility index, an averaged outpatient index (AOI) in accordance with Google’s and Apple’s definitions of the baseline days was calculated. For Google it identifies as the percentual change of the number of outpatients compared to the baseline and is calculated as the median of the number of outpatients for the corresponding days of 2019 for each of the analyzed regions. In equivalence to this, 13 January 2020 was used as a baseline for comparison with Apple data, as this is the baseline period chosen by Apple in the analyses.

Location categories defined by Google are retail and recreation, transit, grocery and pharmacy, workplace, parks and residential. With a view to assess only leisure-related mobility patterns a mobility index (MI_L_) that only included leisure-related movements was calculated, while excluding essential activities such as grocery shopping or visits to the pharmacy (Eq. 1). We also excluded workplaces due to their variability during the pandemic caused by remote work, and the fact that they were not subject to government restrictions. In order to reflect compliance with movement restrictions, the “residency” location was given a negative weight to indicate that individuals remained at home. It is important to note that when referring to the “mobility index” in this study, specifically the leisure-related mobility index (MI_L_) is meant.

$$MI_{L}=\frac{1}{4}*\left[CFB\left(rr\right)+CFB\left(pa\right)+CFB\left(ts\right)-CFB\left(res\right)\right]$$

### Formula 1: leisure related mobility index

CFB = percentual change from baseline, rr = retail and recreation, pa = parks, ts = transit station, res = residential

## Statistical analysis

The data analysis was conducted in R (R Core Team 2021, Vienna, Austria) with usage of the following packages: “ggpubr”, [11] “imputeTS”, [12] “janitor”, [13] “lubridate”, [14] “scales”, [15] “tidyverse”, [16] “tsibble” [17]. α = 0.05 was choosen as the threshold for statistical significance. Besides basic descriptive statistical methods, the Pearson correlation between the MI_L_ and the averaged outpatient index of each city and in total was examined. The baseline value for the number of outpatients was equivalently matched to predefined baseline values of Google and Apple each in order to warrant an equivalent comparison. A simple linear regression model has been used to predict the change of the outpatient index dependent on the GPS-based mobility index. For sporadic data gaps the seasonally splitted missing value imputation by interpolation method for imputing missing data was utilized. The total number of missing days was 38 in Graz and 30 in Innsbruck.

## Ethical approval

The study protocol was approved by the ethics committee and does not contain any trials with human participants or animals. The study was performed in accordance with ethical standard laid down in the Declaration of Helsinki (1964).

## Results

### Correlation and linear regression analysis

**Fig. 1:**
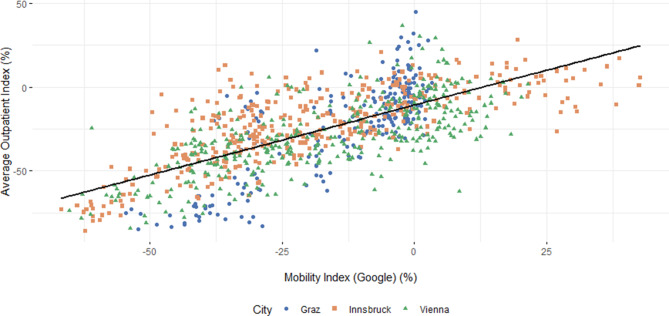
Pearson correlation between average outpatient index and Google MI_L_ for Graz, Innsbruck and Vienna. Scatter plot of the AOI and the Google MI_L_ in all cities (r = 0.70; *p* < 0.001). The graph shows the correlation for Vienna between the Google mobility index and outpatient index (r = 0.70; *p* < 0.001; 95% CI: 0.64–0.75). In Graz a correlation coefficient of r = 0.72, *p* < 0.001, 95% CI: 0.66–0.77 was observed. In Innsbruck the correlation between the AOI and the Google MI_L_ was r = 0.74, *p* < 0.001, 95% CI: 0.69–0.78

This study observed a moderate to high correlation between the mobility indices (Google r = 0.70; *p* < 0.001; 95% confidence interval, CI: 0.67–0.73; Apple r = 0.64; *p* < 0.001; 95% CI: 0.61–0.67) and the averaged outpatient index of all cities (Fig. 1). Although the correlation is clearly significant, Fig. 1 shows substantial heterogeneity in residuals, with increasing variability for larger averaged outpatient index values, indicating that the prognostic ability is less precise for these higher values. Similar results were observed in the analysis of the mobility indices and the averaged outpatient indices of each distinctive city. City-specific correlations between the mobility indices and the averaged outpatient index are shown in Table 1. Following significant regression equation showing a positive relationship for the average outpatient index (VIE) was found: Average Outpatient Index (VIE) = −15.95341 + 0.75997*MI_L_ (Google) with *p* < 0.01. The adjusted coefficient of determination (r^2^ = 0.48) indicates that 48.2% of the variance in the average outpatient index can be explained by the MI_L_. The test resulted in a significance of *p* < 0.01.

**Table 1:** Pearson correlation between the average outpatient index and the Google and Apple

Mobility index provider	Region	Pearson correlation coefficient (r)	Confidence interval (95%)	*p*-value
Google	Vienna	0.70	0.64; 0.75	*p* < 0.001
	Innsbruck	0.74	0.69; 0.78	
	Graz	0.72	0.66; 0.77	
	All cities	0.70	0.67; 0.73	
Apple	Vienna	0.70	0.65; 0.75	
	Innsbruck	0.67	0.61; 0.72	
	Graz	0.68	0.62; 0.73	
	All cities	0.64	0.61; 0.67	

### Changes in mobility and orthopedic trauma epidemiology

**Fig. 2:**
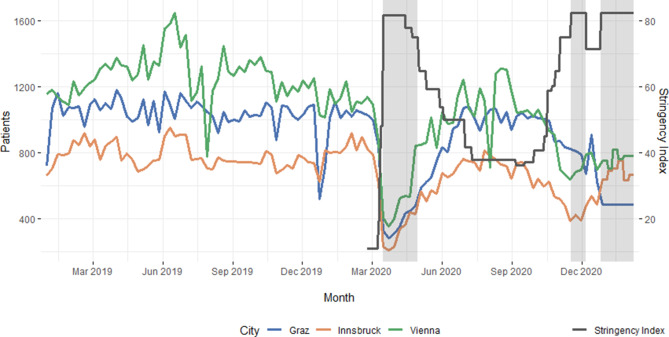
Number of outpatients per week for Graz, Innsbruck and Vienna, shaded periods refer to hard lockdowns. The plots show the number of average outpatients per week per city. There was a total reduction in average patient numbers per day ranging between −61 and –64% during the first lockdown. As measures were loosened due to decreasing infection rates, patient numbers admitted to orthopedic and trauma surgery departments rose slowly and reached their peak in August and September 2020. A further decline of −24 and –46% was recorded throughout the second lockdown. During the third lockdown a decrease in the range of −18 and –32% was observed. The decline in patient numbers in Vienna in August are due to restricted opening of the department due to renovation works

A cumulative reduction of the total outpatient numbers during the period of the first lockdown was observed. A reduction of daily average outpatient numbers of 54.91 (−64%) in Graz, 44.78 (−61%) in Innsbruck (IBK) and 70.89 (−62%) in VIE was recorded. The lowest decrease during a lockdown was monitored during the third lockdown. Figure 2 shows the development of the average outpatient numbers over time and Table 2 displays the changes in trauma patient numbers per day during each lockdown.

**Table 2:** Number of outpatients per lockdown per day and differences to prepandemic era

City	Gender	1st Hard lockdown		2nd Hard lockdown		3rd Hard lockdown	
		16/03/2019–30/04/2019	16/03/2020–30/04/2020	17/11/2019–06/12/2019	17/11/2020–06/12/2020	26/12/2019–07/02/2020	26/12/2020–07/02/2021
		AO/d	AO/d (diff%)	AO/d	AO/d (diff%)	AO/d	AO/d (diff%)
Graz	Female	78.9	28 (−64%)	80.7	60.8 (−25%)	73.8	56.8 (−23%)
	Male	73.9	26.9 (−64%)	69.9	53.9 (−23%)	65.1	37.7 (−42%)
	*Total*	*152.8*	*54.9 (−64%)*	*150.6*	*114.6 (−24%)*	*138.9*	*94.5 (−32%)*
Innsbruck	Female	50.3	20 (−60%)	46.3	26.7 (−42%)	54.7	46.3 (−15%)
	Male	65.4	24.7 (−62%)	59.4	30.2 (−49%)	62.3	50.1 (−20%)
	*Total*	*115.7*	*44.8 (−61%)*	*105.7*	*56.8 (−46%)*	*117*	*96.4 (−18%)*
Vienna	Female	87.4	33.1 (−62%)	84.5	47.8 (−43%)	77	55.4 (−28%)
	Male	101.1	37.8 (−63%)	85.6	47.5 (−45%)	80.9	53.2 (−34%)
	*Total*	*188.5*	*70.9 (−62%)*	*170.1*	*95.3 (−44%)*	*157.9*	*108.6 (−31%)*

*AO/d* average number of outpatients per day, *per diff%* percentual difference to the same period of the previous year

**Fig. 3:**
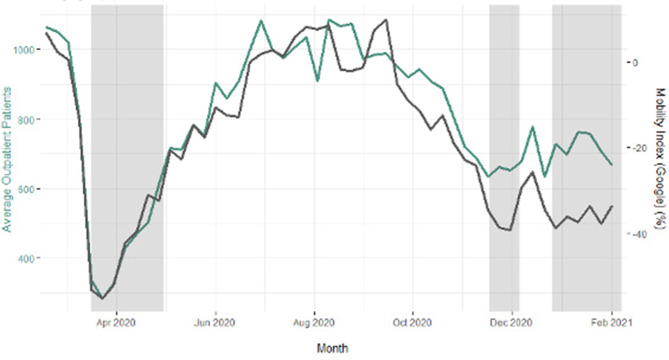
Average number of outpatients per week for all cities vs. average Google MI_L_ for all cities. Shaded periods refer to hard lockdowns. The maximum decrease of movement and patient numbers in all observed federal states is recorded during the first lockdown, indicating that people’s compliance to the measures also declined as time passed

Google’s community mobility data reports show a decrease in mobility ranging between −40 and–48% during the first lockdown. In the period of the 2nd lockdown, a reduction of −29 and –43% was observed. During the third lockdown the mobility index ranged from −31 and –37%. The analysis of Apple’s data also showed the highest decline during the first lockdown, with a reduction up to −75%. The decrease during the second lockdown ranged from −39 to 64% and during the third lockdown from −35 and –59%. As neither Google nor Apple provide absolute numbers in their mobility reports, only the percentual change to the baseline value can be stated. Figure 3 shows the changes in mobility in comparison to the changes in trauma outpatient numbers for all cities.

## Discussion

This study analyzed the association between GPS-based mobility data and trauma patients to validate geography-based mobility analyses as a tool for assessing orthopedic trauma patient loads. Moderate to high correlations between the MI_L_ and the number of outpatients were found. This indicates that GPS-based mobility data has the potential to serve as a prediction tool for the amount of trauma patients. These findings improved our understanding of the relationship between mobility patterns and trauma patients, and in turn, the ability to predict the number of trauma patients in the future. This could lead to improved resource planning, public health policy, and cost-effectiveness, thereby enhancing patient care and reducing the burden on orthopedic trauma departments, especially during challenging times such as the COVID-19 pandemic. Furthermore, it could be stated that hard mobility restrictions led to a decrease in mobility and trauma patient numbers, albeit with incremental duration a decreasing compliance to the measures was observed. Therefore, one can conclude that at least for a certain time period movement restrictions are a suitable method to reduce trauma patient numbers in emergency situations such as pandemics but a loss of effectiveness over time is to be expected.

### The relationship between GPS mobility data and trauma patients

A moderate to high correlation between both MI_L_ and the cumulative trauma patient numbers in all cities were observed. The correlations of each distinctive city were equivalently strong. This means that the decrease of the population’s movement is associated with a decrease in trauma patients and vice versa. As similar results using both mobility indices (Google and Apple) has been calculated, the results of this study suggest that the high correlation is not bound to a specific tool or provider. Hence, mobility data have the potential to be used for predictions of trauma patient numbers. This would be of advantage in multiple respects. Besides being prepared for future infection waves and pandemics, it could be useful as a prospective basis for estimating how much personnel, materials or beds will be needed in specific time periods. A simple linear regression model showed that when the Google mobility index increases by 0.76 units, it is expected that the outpatient index will increase by 1 unit. The F‑test shows that the regression model is significant (*p* < 0.01), meaning that the relationship between the two variables is not due to chance. This suggests that the MI_L_ is a good predictor of the average outpatient index, and that changes in the Google mobility index can be used to estimate changes in the number of trauma patients.

There are multiple other investigations suggesting that GPS-based mobility data are applicable to assess a population’s general mobility patterns and their adherence to the imposed restrictions [7]. Moreover, there is evidence that mobility patterns show correlations with disease spread and infection rates [18]. For example, Periyasamy et. al. analyzed the relationship between the doubling time of the infection rate and Google location data in India [19]. They found a strong negative correlation of mobility patterns and the COVID-19 doubling time and created a model to predict the doubling time based on Google mobility data. Lami et. al. analyzed the relationship between growth ratio and mobility patterns and came to the same conclusion [20]. Scott et. al. found a strong correlation between the number of trauma patients and the Google mobility indices [21]. To our knowledge this is the only other study evaluating the correlation between the Google mobility index and the number of trauma patients and differs from our study in several key ways [21]. Our study provides a more comprehensive and focused examination of this relationship in Austria using data of two providers over a longer time period and hence advances the understanding of this topic.

### Changes in epidemiology and mobility

The governmental imposed mobility restrictions achieved their intended impact on the population’s mobility as well as on the trauma patient numbers. This study found the highest decrease during the first lockdown. These accounted for −47% decrease in mobility and −64% in outpatients compared to the same time period in the previous year. During the subsequent lockdowns, reductions were recorded as well, albeit these were less pronounced. This observation is in accordance with other studies that investigated the development and dynamics of orthopedic and trauma epidemiology [22]. An additional factor that could have added up to the strong reduction in patient numbers is the fear of getting infected in the hospital [23]; however, this explanation might mostly apply to ambulant patients with less severe injuries. Our findings let us conclude that movement restrictions are effective tools for curbing trauma patient numbers. Especially the initial hard constraints yielded the greatest effect. Further repetitive restrictions of long durations led to a decrease in adherence, which was reflected in higher GPS-based mobility. Consequently, this led to higher numbers of patients admitted to trauma units. Studies on effectiveness of governmental containment measures after the first wave suggested that the most effective way to stop the virus spread are hard lockdowns with restriction of social gatherings and movement restrictions [24]; however, it should be remarked that these measures also have damaging effects on economics, mental and physical health and security. They only present as an appropriate approach for a limited time period [25]. This is also reflected in our data, as there seems to be a decreasing compliance to the restrictions. Thus, it should be queried if the risk-benefit ratio of lockdowns still yields positive results regarding reduction of accidents in future studies.

## Limitations

A major limitation of this study is its retrospective character and thus unavoidable scattered data gaps that were filled using linear interpolation. Furthermore, data gaps in Google’s and Apple’s mobility data can occur when the company’s privacy and quality threshold cannot be met. Moreover, seasonality is not incorporated in the baseline days, therefore mobility indices can vary depending on weather changes for example. Google and Apple users represent a sample that does not necessarily allow generalizability to the entire Austrian population (especially the older age groups) even though a broad population is covered. The general number of smartphone users in Germany aged 14–59 years amounts to over 90%. Over 80% of the 60–69-year-olds use a smartphone as well as at least over 52% of those aged 70+ years. The Austrian populace shows a similar distribution. This statistic shows that the great proportion of the population possesses and uses a smartphone and is therefore captured in the GPS-based mobility analysis. Marginalized groups like under 14-year-olds and people well above 70 years may not be depicted ideally in the mobility data. As the vast majority of the populace is depicted in these data, a general mobility trend can be gauged, nonetheless.

## Conclusion

This study has found that there is a moderate to high correlation between GPS-based leisure-related mobility data and the number of trauma patients and that this relationship is provider independent. Especially during the COVID-19 pandemic orthopedic trauma departments faced several challenges. Therefore, estimating the number of trauma patients with a model built upon our study findings could provide more efficient and effective care. In further instance this could help with resource planning, improve public health policy, and enhance cost-effectiveness. A further observation of our study was that as the COVID-19 pandemic progressed, compliance with mobility restrictions decreased, leading to increased mobility and an increase in the number of trauma patients. These findings highlight the importance of understanding the connection between mobility patterns and trauma patients in order to provide better care and mitigate the impact of future pandemics or similar demanding situations.

## Data Availability

The datasets used and/or analyzed during the current study are available from the corresponding author upon reasonable request.
